# Case Report: Phenotypic heterogeneity within an NF1 family: assessment of the pathogenicity of a *de novo* c.6640dupA shift mutation and a splice variant with an epilepsy phenotype

**DOI:** 10.3389/fnins.2025.1604771

**Published:** 2025-07-09

**Authors:** Sijing Ren, Yanling Wang, Xinhua Tong, Xiaoyu Wu, Huan Yan, Qing-Xia Kong

**Affiliations:** ^1^Department of Neurosciences, Affiliated Hospital of Jining Medical University, Jining, China; ^2^The Second Clinical Medical College, Shandong University of Traditional Chinese Medicine, Jinan, China; ^3^Key Laboratory of Basic and Clinical Transformation of Epilepsy Pathogenesis, Jining, China

**Keywords:** neurofibromatosis type 1, *NF1* gene mutation, seizures, rare diseases, genotype–phenotype analyses

## Abstract

**Purpose:**

Neurofibromatosis type 1 (NF1) is a complex autosomal dominant disorder with wide variability in its clinical presentation, rate of progression, and severity of complications. The majority of patients have point mutations, but no specific mutational hotspots have been identified. The aim of the present study was to better understand the genotypic and phenotypic characteristics of NF1 by conducting a detailed analysis of a single case, from genetic diagnosis to the exploration of underlying mechanisms.

**Methods:**

The study included an 11-year-old girl with epilepsy who presented to our hospital in 2021. Clinical data of the patient and her family members were collected, and peripheral venous blood samples were analyzed for causative genes using whole-exome sequencing. The identified genes were validated using Sanger sequencing, and alterations in the tertiary structural physicochemical properties of the mutant proteins were analyzed using AlphaFold2 bioinformatics software.

**Results:**

We identified two mutation sites in the *NF1* gene of the patient. A heterozygous c.6640dupA mutation that leads to an amino acid shift (p.R2214Kfs*7) was inherited from the mother. This mutation site is new and has not been previously reported. Despite having the same mutation, the patient had seizures with a completely different clinical presentation than that of her mother, who had had intracranial tumors. This finding prompted us to consider potential differences in genotype–phenotype correlations in this NF1 family line. The other identified mutation, a heterozygous c.7395-3C > G mutation that results in amino acid splicing, was inherited from the patient’s father. Although the father carried the mutation, he did not have any related manifestations. We therefore suspected that the mutation at this locus was not pathogenic, a suspicion we confirmed in the patient’s brother.

**Conclusion:**

In the present study, we not only identified previously undescribed *de novo* mutations in *NF1* but also contributed to a broader understanding of the NF1-related *NF1* gene profile. Our findings have implications for the molecular diagnosis of the disease and the development of effective therapeutic approaches.

## 1 Introduction

Neurofibromatosis type 1 (NF1) is a complex autosomal dominant disorder that affects multiple organ systems. It occurs in 1 in 2,500–3,000 individuals worldwide and varies widely in its clinical presentation, rate of progression, and severity of comorbidities (Uusitalo et al., 2015). NF1 is one of the three diseases described under the broad definition of “neurofibromatosis.” The other two are neurofibromatosis type 2 and neurocysticercosis, which are clinically and genetically distinct from NF1 (Cimino and Gutmann, 2018). Almost all patients with NF1 develop pigmented lesions (café au lait spots, skin fold freckles, and Lissy nodules) and dermal neurofibromas. Some individuals also develop skeletal abnormalities (scoliosis, tibial pseudoarthrosis, and orbital dysplasia), brain tumors (optic nerve gliomas and glioblastomas), peripheral nerve tumors (spinal cord neurofibromas, plexiform neurofibromas, and malignant peripheral nerve sheath tumors), headaches, seizures, learning disabilities, attention deficits, and social and behavioral problems. These complications progress throughout the lifespan of an individual and can negatively affect their quality of life (Gutmann et al., 2017). Currently, there is no definitive treatment for NF1, and clinical management is usually limited to monitoring the condition and providing symptomatic care, which often involves surgery to address specific complications.

NF1 is caused by heterozygous mutations in the *NF1* gene, which is a large gene located on chromosome 17q11.2 and has a total length of approximately 350 kb. It contains 57 exons, and there are also 13 homozygous *NF1* pseudogenes, which complicate its molecular analysis (Cawthon et al., 1990). *NF1* encodes neurofibromin, which is a tumor suppressor protein consisting of 2,818 amino acids. Neurofibromin is expressed in many cell types, including neurons, glial cells, immune cells, endothelial cells, and adrenal medullary cells. Notably, it may have different functions in different cell types (Banerjee et al., 2016). Neurofibromin is a negative regulator of the Ras GTPase-activating protein. In NF1, the reduction or loss of neurofibromin function stimulates transcription, as well as cell growth and proliferation, through hyperactivation of the Ras cascade (Denayer et al., 2008). Consequently, the loss of neurofibromin has a wide range of pathological consequences, including the formation of pigmented lesions, tumors, and skeletal abnormalities. Although genetically engineered mouse models of NF1 have helped elucidate some of the mechanisms underlying these abnormalities, no single mouse model exhibits all (or even most) of the features of NF1. Importantly, irrespective of population distribution, 50% of NF1 cases are familial. Of these, 90% of variants are inherited from paternal chromosomes, and the remaining cases are caused by *de novo* mutations in *NF1*. To date, more than 2,600 mutations have been identified, the majority of which are point mutations; no mutational hotspots have been found (Tsipi et al., 2018). The large size of *NF1* (57 exons), the relative lack of mutational hotspots, the diversity of causative mutations, and the lack of clear genotype–phenotype correlations contribute to the challenges of conducting genetic screening for NF1 in clinical settings.

In this study, we report two *de novo* compound heterozygous mutations in *NF1*. One was a heterozygous c.6640dupA mutation, resulting in a shifted amino acid (p.R2214Kfs*7). This mutation was inherited from the mother and was considered the causative gene. It is a new mutation site that has not been previously reported, and it had a completely different clinical phenotype in the mother and daughter. The other mutation was a heterozygous c.7395-3C>G mutation, resulting in amino acid splicing. This mutation was inherited from her father, who carried the mutation but did not have any related manifestations. We therefore suspected that the mutation at this locus was not pathogenic and confirmed the conjecture in the patient’s brother.

## 2 Materials and methods

### 2.1 Clinical characteristics

The patient was a 14-year-old girl who had experienced paroxysmal headaches since the age of 5 years. She was admitted to the hospital at 11 years of age because of developmental regression and aggravation, which was characterized by personality changes, panic attacks with limb shaking, involuntary laughter, prolonged episodes of slurred speech, and logic normalized after remission but speech still slurred. Her condition gradually worsened to the extent that it affected her daily life and made it impossible for her to attend school. When the patient was 14 years old, she started experiencing episodes of unconsciousness without clear triggers, which were mainly characterized by the sudden onset of unconsciousness, an inability to call out, shaking of the head and upper limbs, or staring without twitching of the limbs. These episodes lasted for more than 10 s and occurred a few times per day. In addition, she sometimes felt pain at the top of her head but was unable to clearly describe the nature of the pain.

The patient took oral levetiracetam (250 mg, twice daily) but still experienced 2–3 seizures per day. After switching to perampanel (6 mg, nightly), the convulsions ceased, but she felt irritable and fearful. When treated with oxcarbazepine (300 mg, twice daily), she experienced frequent headaches. She was then treated with zonisamide (50 mg, twice daily) but her appetite diminished and she ate little, after which she stopped speaking; she therefore discontinued taking zonisamide. She is currently taking levetiracetam (125 mg, twice daily) plus oxcarbazepine (300 mg, twice daily), with good treatment effects.

The patient was born at term via cesarean section, with no history of hypoxia at birth and a normal Apgar score. The patient’s father and brother are healthy, and her parents are not consanguineous. Prior to disease onset, she had normal physical and intellectual development, consistent with that of her peers. Her mother had had a hemangioma on her mandible since childhood, which gradually enlarged. When the mother was 20 years old, she began experiencing dryness, fatigue, discomfort, swelling, and pain in the left side of her eyes, accompanied by decreased vision. She was diagnosed with “glaucoma in the left eye and cataract” and underwent surgical treatment; however, she is currently blind. She was also diagnosed with an “intracranial tumor” and underwent surgical treatment, after which her symptoms disappeared.

## 3 Results

### 3.1 Physical examination

The patient was conscious, mentally available, and generally responsive. She had normal development, good nutrition, and a scattered distribution of café au lait spots of different sizes on her trunk (Supplementary Figures 1A,B). There was no deformity of the spine, her limbs moved freely, her joints were normal, and her lower limbs were not swollen. The muscle strength of her limbs was grade V, her muscle tone was normal, and her abdominal wall reflexes were normal.

### 3.2 Imaging

**Figure 1:**
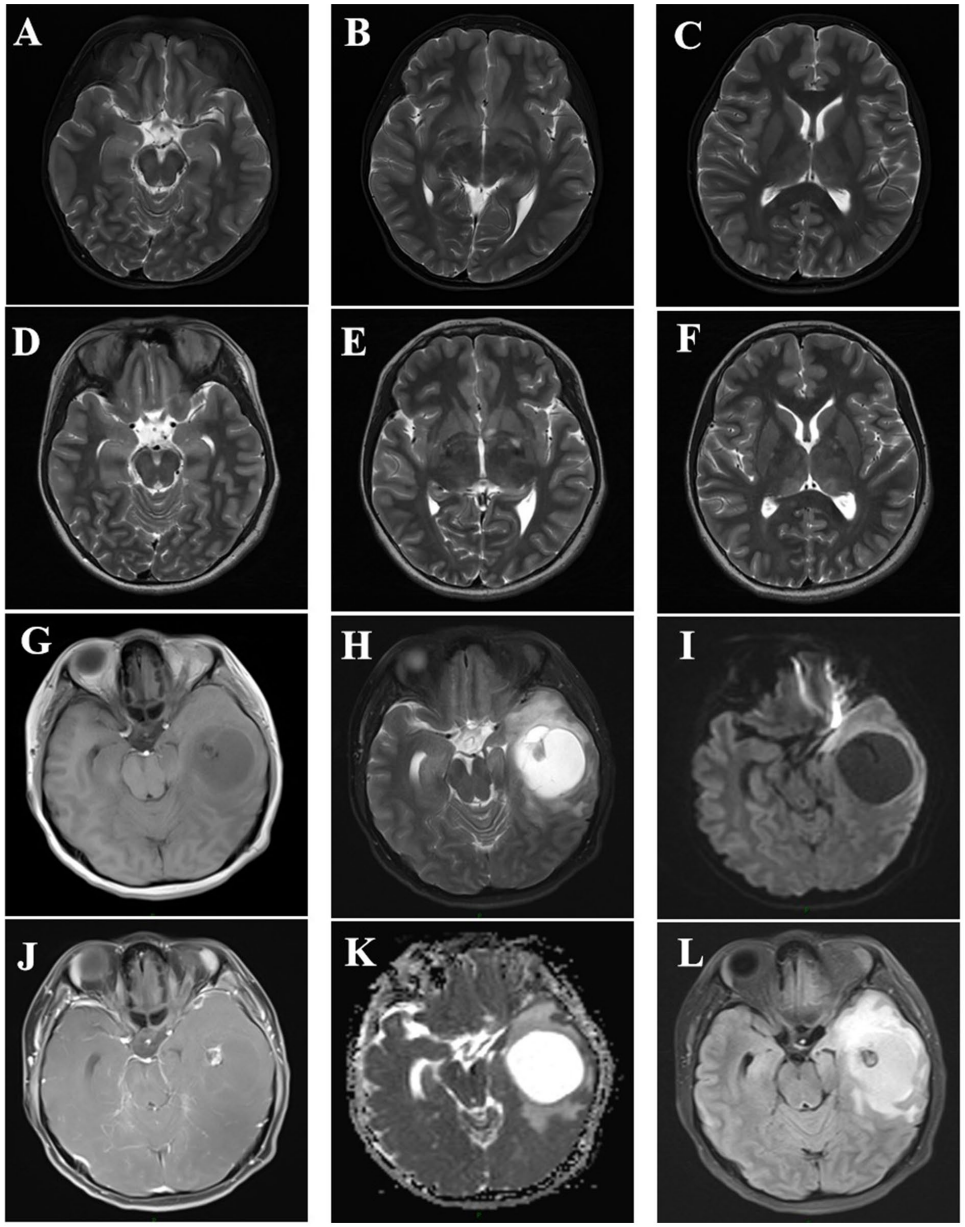
**(A–C)** Cranial magnetic resonance imaging of the patient at 11 years of age, showing patchy T2-weighted imaging (T2WI) high signals with fuzzy boundaries in the bilateral basal ganglia, thalamus, brainstem, and bilateral cerebellar hemispheres, with no diffusion limitation on diffusion-weighted imaging (DWI). **(D–F)** Cranial magnetic resonance results of the patient at the age of 14 years after a seizure, showing findings consistent with the previous results (**A–F** correspond to the same levels). **G–L** Cranial magnetic resonance imaging of the patient’s mother at the age of 31 years, showing changes indicating a solid cystic mass in the left temporal lobe. **A** T1-weighted imaging (T1WI), **B** T2WI, **C** apparent diffusion coefficient (ADC), **D** T1WIC+, **E** DWI, and **F** fluid-attenuated inversion recovery (FLAIR).

At 11 years of age, transcranial Doppler ultrasound revealed increased blood flow velocity in the left internal carotid artery system, and intracranial vascular malformation was suspected. Cranial magnetic resonance imaging (MRI) revealed the following findings. T2-weighted imaging showed patchy high signal intensities in the bilateral basal ganglia, thalamus, brainstem, and bilateral cerebellar hemispheres, with fuzzy boundaries. Diffusion-weighted imaging (DWI) showed no diffusion limitation and no significant signal abnormalities in the rest of the brain parenchyma, and the ventricular system did not have any significant deformation (the midline structure was centered). Magnetic resonance angiography revealed that the intracranial segment of the bilateral vertebral arteries had a tortuous course; the basilar artery ring was intact; the courses of the bilateral internal carotid arteries, basilar arteries, and bilateral anterior cerebral, middle cerebral, and posterior cerebral arteries and their branches were normal, with no obvious focal thickening or thinning; and there were no abnormal blood vessel clusters (Figures 1A–C). At the age of 14, MRI demonstrated patchy T1 signals in the bilateral basal ganglia, thalamus, corpus callosum, brainstem, and bilateral cerebellum. Both hemispheres showed patchy low signals on T1-weighted imaging (T1WI) and high signals on T2-weighted imaging (T2WI), with no diffusion restriction observed on diffusion-weighted imaging. The ventricles, cerebral pools, fissures, and sulci were symmetrical, normal in size and morphology, and centered on midline structures. The size, morphology, and signal of the pituitary gland did not show any clear abnormalities (Figures 1D–F). Other imaging findings appeared normal.

### 3.3 Video electroencephalogram (EEG) monitoring

**Figure 2:**
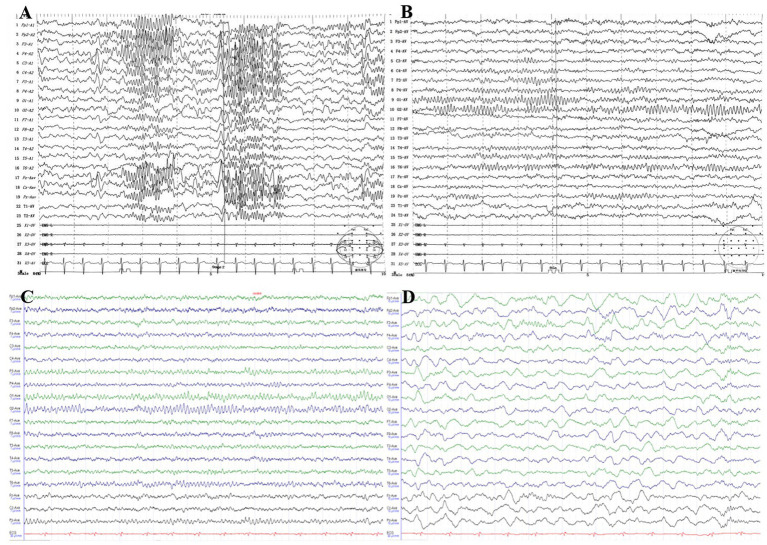
**(A,B)** Video EEG of the patient at 11 years of age, showing abnormal focal bilateral central, parietal, and central midline areas across all wake–sleep phases. The spike wave, spike slow wave, spike slow wave/spike slow wave sleep phase were prominent. **(C,D)** Video EEG of the patient at 14 years of age, showing a limbic state: alpha dissociation and an increase in slow waves were observed in the sleep phase, top waves and sleep spindles appeared as expected, and a slow wave sleep phase was present.

At the age of 11 years, video EEG showed abnormal childhood EEG patterns, with focal bilateral central, parietal, and central midline areas across all phases of wake–sleep; spike waves, sharp waves, spike slow waves were observed, and the spike slow wave sleep phase was significant (Figures 2A,B). At the age of 14 years, video EEG showed a borderline state: alpha dissociation was observed in the sleep phase, slow wave activity was increased, the top wave and sleep spindles appeared as expected, and a slow wave sleep phase was noted (Figures 2C,D). The patient was diagnosed with symptomatic epilepsy.

### 3.4 Laboratory tests

Urine organic acid measurements and blood genetic metabolism tests were unremarkable at 11 years of age; no other laboratory tests showed abnormalities.

### 3.5 Genetic testing

**Figure 3:**
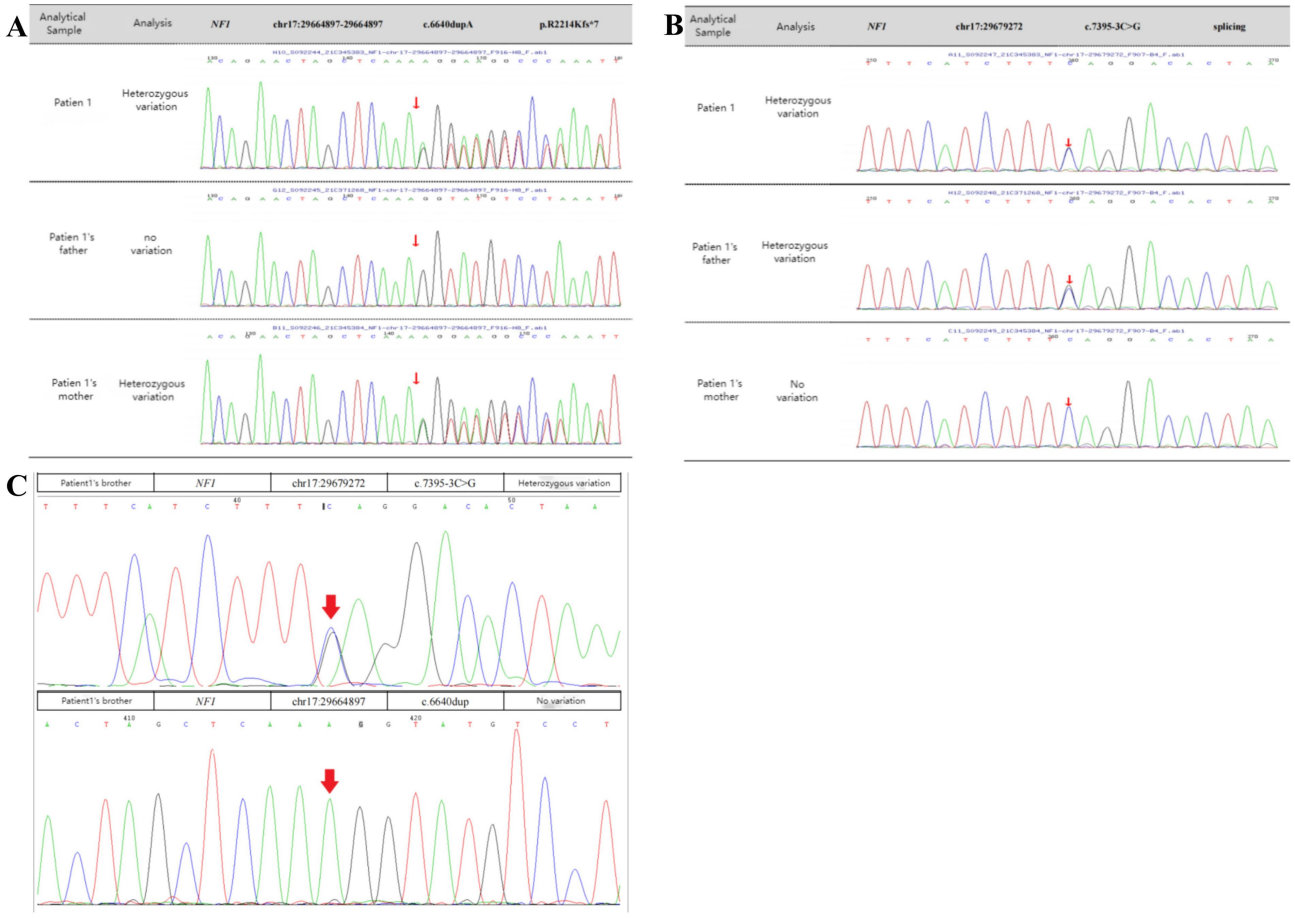
Sanger sequencing confirmation. **(A,B)** The patient had two heterozygous mutations in *NF1* (arrows). One was a heterozygous c.6640dupA mutation, resulting in an amino acid shift (p.R2214Kfs*7), a pathogenic variant that may result in loss of gene function. The variant at this locus was inherited from the mother, and there was no variant at this locus in her father. The other was a heterozygous c.7395-3C > G mutation, resulting in amino acid splicing. The variant at this locus was inherited from the father, and there was no variant at this locus in her mother. **(C)** Genetic testing of the specific locus in the patient’s brother. The patient’s brother had the heterozygous c.7395-3C > G mutation from their father but did not have the heterozygous c.6640dupA mutation.

When the patient was 11 years old, 2 mL of peripheral venous blood was drawn from the patient and her parents. Genomic DNA was extracted from the blood using standard whole-exome sequencing methods, which identified two heterozygous mutations in the *NF1* gene of the patient. The first was a heterozygous c.6640dupA mutation, resulting in an amino acid shift (p.R2214Kfs*7). This mutation is pathogenic and may result in loss of gene function. The mutation at this locus was inherited from the patient’s mother, while the father had no mutation at this locus. The second was a heterozygous c.7395-3C>G mutation, resulting in amino acid splicing. The variant at this locus was inherited from the patient’s father, and her mother had no variant at this locus (Figures 3A,B). The patient was therefore diagnosed with NF1. In addition, since the patient’s father showed no related manifestations, we suspected that the c.6640dupA mutation was not pathogenic. Therefore, we investigated the mutation by direct sequencing in the patient’s 11-year-old brother. The heterozygous c.7395-3C>G mutation was also identified in the patient’s younger brother (Figure 3C).

## 4 Discussion

**Figure 4:**
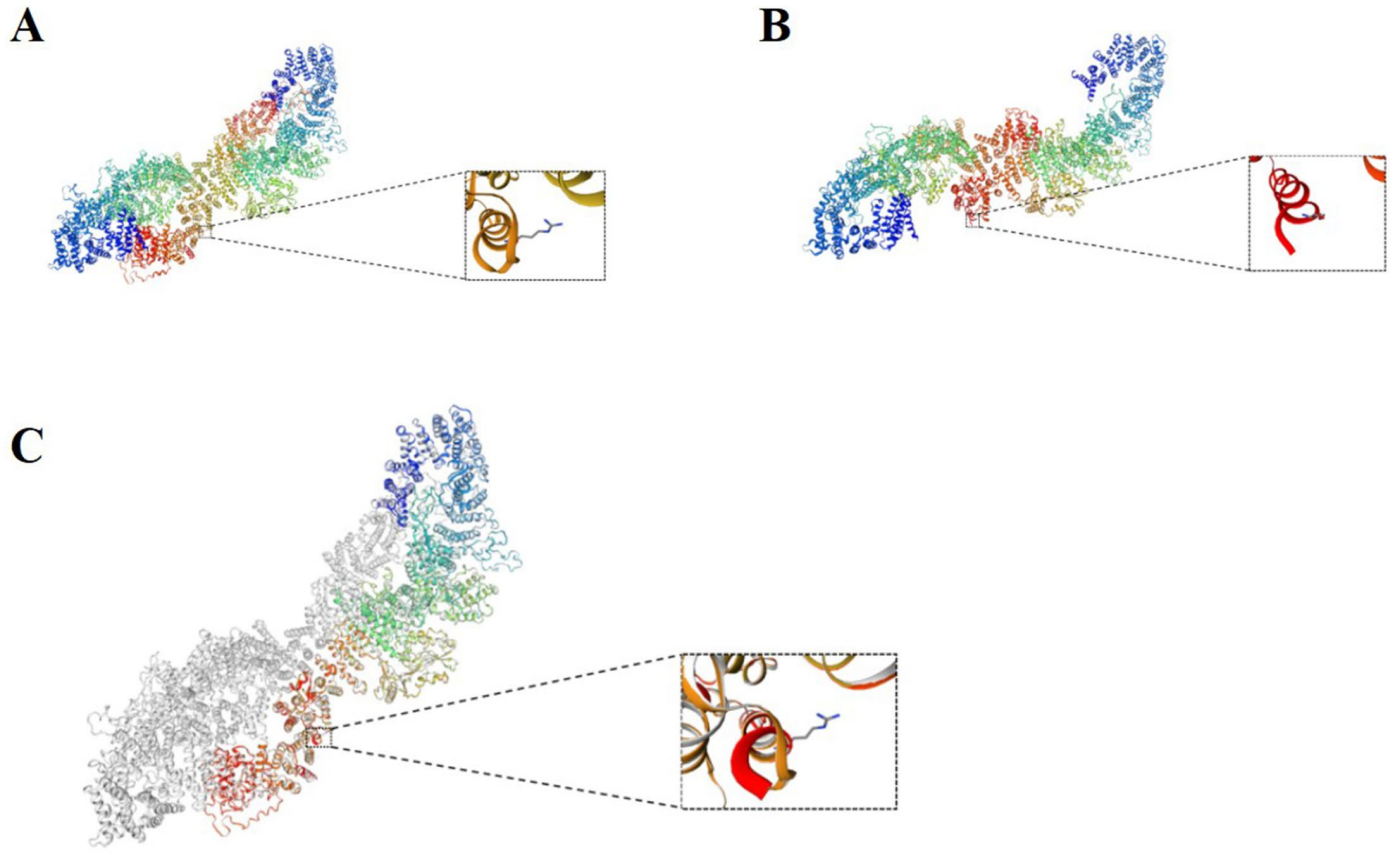
**(A)** Normal protein tertiary structure of the *NF1* gene. **(B)** Protein tertiary structure of the c.6640dupA heterozygous mutation of the *NF1* gene. **(C)** Comparison between the normal *NF1* gene and the mutation.

NF1 is an autosomal dominant disorder, meaning all individuals with germline *NF1* mutations will have the disease. However, patients may exhibit extreme variability in clinical features, even among individuals from the same family with the same germline *NF1* mutation (Gutmann et al., 2017). In this regard, we report a patient and her family as characteristic and representative cases of NF1. In our patient, we used genetic testing to identify two mutation sites in *NF1*. One of these was a heterozygous c.6640dupA mutation, resulting in an amino acid shift (p.R2214Kfs*7); this pathogenic mutation may lead to loss of gene function. The mutation at this site was inherited from her mother, and there was no mutation at this site in her father. Furthermore, this is a new mutation site that has not been previously reported in the literature. Interestingly, the patient and her mother had significantly different clinical features, except for the presence of multiple café au lait spots scattered over the body. The patient presented with seizures accompanied by occasional pain of an indescribable nature at the top of her head. Her mother had a greater number of café au lait spots of varying sizes scattered over her trunk (Supplementary Figures 1C,D), as well as a hemangioma on the mandible that had been present since childhood (Supplementary Figure 1E). At the age of 31 years, a cystic solid occupancy in the left temporal lobe of the cranium was detected in the patient’s mother (Figures 1G–L); it was diagnosed as an intracranial tumor and treated surgically. Postoperative pathology revealed a microscopically visible vascular malformation in the left temporal lobe (Supplementary Figure 2). Although they both had the same mutation at the same locus of *NF1*, the patient had seizures, whereas the patient’s mother did not have seizures and presented with the more common fibromatosis complications of intracranial tumors and vascular malformations. This is the most noteworthy finding of our case. Nonetheless, it can still be argued that the *NF1* mutation may lead to a loss of neurofibromin protein function from the mutant allele. We therefore explored this possibility using protein tertiary structure prediction (Figure 4). Our findings suggest that there is no association between the type and location of the *NF1* mutation and the NF1 phenotype and seizures, which is an interesting and unique result. As NF1 is completely exogenous, we analyzed the parents and other high-risk members of the patient’s family (47 relatives). This analysis revealed a significant family history on the maternal side (Supplementary Figure 3) and a high degree of clinical variability within the same family, which has not been observed in previous studies. However, due to certain factors, we were unable to perform a comprehensive examination and genetic testing on all 47 relatives. Therefore, the major limitation of our study is the inability to perform further genetic epilepsy testing across the entire extended family and confirm the presence of additional genetic alterations related to susceptibility.

The available literature on NF1 indicates that genotype–phenotype correlation differences in NF1 have long been observed in members of the same family, although the exact cause for this remains unknown (Hébert et al., 2024). We believe that this inconsistency between the genotype and phenotype may be explained by environmental factors and modifier genes or a combination of both. Some studies have noted that the TATA-binding protein-like factor (TLF) is a functional transcription factor that activates RNA polymerase II transcription at a subset of promoters. The TLF increases transcription from the endogenous *NF1* promoter at the same major start sites as those naturally occurring *in vivo*. The TLF also binds to and increases the rate of transcription from an *NF1* promoter-dependent reporter construct. In addition, purified TLF–transcription factor IIA binds to the *NF1* promoter *in vitro*, and the TLF–green fluorescent protein associates with an *NF1* promoter fragment *in vivo* (Chong et al., 2005; Pasmant et al., 2012). Moreover, approximately 50% of NF1 patients are affected by *de novo* variants, and germline chimeras have been identified in some patients (Ko et al., 2013; Du et al., 2022). Our findings expand the spectrum of NF1 genotypes and phenotypes induced by *NF1* mutations and may be useful for the screening and genetic diagnosis of NF1.

The patient had another heterozygous mutation, c.7395-3C>G. The chromosomal location of the mutation was chr17:29679272, and the transcript exon was NM 000267, exon 50. The c.7395-3C>G mutation in *NF1* is located in the 3′ splice site region of intron 48 (position −3 of the polypyrimidine bundle). This mutation (C>G) disrupts the structure of the polypyrimidine bundle and severely impairs the binding of the splicing factor U2AF65, leading to the failure of the normal splice acceptor function of intron 48. This results in amino acid splicing, and this mutation was inherited from her father. According to the American College of Medical Genetics and Genomics guidelines, this variant was initially determined to be clinically unspecified (uncertain) PM2. The ClinVar database classified the pathogenicity of this locus as having uncertain significance, associated with hereditary cancer-predisposing syndrome and neurofibromatosis type 1. This mutation site has been previously reported in the literature (Palma Milla et al., 2018). c.7395-3C>G is an intronic variant that affects a non-highly conserved residue across vertebrates, and we were unable to predict protein structural changes. However, the Human Splicing Finder bioinformatic tool (Desmet et al., 2009) predicts an alteration of the wild-type acceptor site, most probably affecting splicing. In addition, because the patient’s father showed no positive manifestations, we suspected that the mutation at this locus was not pathogenic. We therefore performed direct sequencing on the patient’s brother and found that he had also inherited the heterozygous c.7395-3C > G mutation from his father; the patient’s brother also showed no related manifestations at that time. Given that negative results do not exclude mutations, especially in non-coding portions of the gene, we cannot make any definitive conclusions; however, our case provides a basis for such speculation. Accurate splicing results need to be confirmed by other experimental means, such as reverse transcription polymerase chain reaction, RNA sequencing, or the Minigene assay (Morbidoni et al., 2021).

Epilepsy is a well-recognized neurological complication in patients with NF1, with an estimated prevalence of 4–13% (Nix et al., 2020). This value is markedly higher than the 1–2% reported in the general population, indicating that seizures are more common in NF1 patients than in the general population. Individuals with seizures are more likely to have inherited NF1 from their mothers (*p* = 0.001), consistent with our patient’s case. Their seizures are usually focal and primarily associated with intracranial tumors or structural abnormalities, including hippocampal sclerosis, focal cortical dysplasia, and multiple cerebellar gyrus malformations (Barba et al., 2013). However, the etiology of seizures in patients with NF1 is unclear; it has been suggested that the brains of NF1 mice exhibit hyperexcitability caused by altered and dysfunctional *γ*-aminobutyric acid signaling and many ion channels. This hyperexcitability is considered a possible direct cause of NF1 seizure development, although the evidence remains insufficient (Moutal et al., 2017; Stafstrom et al., 2017). Some animal findings also suggest that the mammalian target of rapamycin (mTOR) pathway plays an important role in epileptogenesis in NF1(Citraro et al., 2016). mTOR is constitutively activated in NF1, and neurofibromin loss leads to Ras hyperactivation, which subsequently leads to hyperactivation of both RAF/mitogen-activated protein kinase /extracellular signal-regulated kinase and phosphoinositide 3-kinase/mTOR pathways (Johannessen et al., 2005). Therefore, neurons in the central nervous system of NF1 patients may be hyperexcitable, and this plays a potential role in subsequent seizure susceptibility. Considering the two aforementioned hypotheses, together with our finding that the patient had unstructured seizures in the context of NF1, it is possible that NF1 itself is the cause of the EEG dysfunction and the possible presence of other genetic predisposition factors. Maternal inheritance of NF1 is also considered a non-negligible predisposing factor for seizures. Nonetheless, whether this heterozygous c.6640dupA mutation, which results in a code-shifting amino acid (p.R2214Kfs*7), is involved in epileptic pathogenesis remains to be confirmed in a larger number of cases.

Neuroimaging during seizures revealed new structural abnormalities in 21% of NF1 patients studied using MRI. These findings suggest that all people with NF1 and new seizures should undergo MRI even if prior neuroimaging has been normal (Ostendorf et al., 2013). However, these findings are inconsistent with our reported case. No seizures were detected at the age of 11 years, at which time the MRI findings showed multiple abnormal signals in the brain parenchyma, consistent with the MRI manifestations of neurofibromatosis. In addition, MRI after a seizure at the age of 14 years showed the same imaging findings, with no new structural changes. This suggests that the new structural abnormalities identified after seizures may be coincidental and not related to NF1 or seizures. There is also controversy regarding whether the observed T2 high signals in the basal ganglia, thalamus, cerebellum, and brainstem are associated with an increased risk of seizures, especially in medial temporal lobe epilepsy (Hsieh et al., 2011; Labate et al., 2010). Therefore, the etiological relationship between T2 high signals and seizures, as well as the distinction between pathological and characteristic T2 high signals in some cases, remains unclear and requires further investigation.

Although the pathophysiological processes underlying the increased risk of seizures in NF1 patients are not fully understood, multiple etiologies appear to point to the hyperactivation of brain excitability. It is estimated that approximately 25% of NF1 patients have abnormal EEG (Ostendorf et al., 2013; Khair et al., 2022). The majority of seizures are symptomatically focal with or without secondary generalized features, although generalized seizures have also been reported (Kulkantrakorn and Geller, 1998; Omana Suarez et al., 2025). This is consistent with our present findings. However, there are no significant correlations between the presence or location of intracranial tumors and epileptic symptomatology or EEG localization with respect to the epileptic EEG features of patients with NF1. In addition, previous studies on children with epilepsy in the general population have reported that epilepsy is controlled with a single antiepileptic drug in 50–70% of patients and that 60% of individuals with NF1 achieve good seizure control with one or no antiepileptic drugs (Korf et al., 1993). However, cohort findings also suggest that individuals with NF1 and epilepsy are often more difficult to treat with monotherapy (Ostendorf et al., 2013). After our patient was diagnosed with an *NF1 de novo* compound heterozygous mutation complicated with more severe seizures, she was first given levetiracetam (250 mg, twice daily), which was not effective. The treatment was then changed to perampanel (6 mg, nightly), and although seizures did not recur, she experienced frequent and obvious episodes of fear. After oxcarbazepine (300 mg twice daily) was added, headaches developed, prompting the addition of zonisamide (50 mg twice daily) to manage the pain. However, the patient subsequently exhibited poor appetite and loss of speech. Zonisamide was then discontinued, and the treatment was changed to levetiracetam (125 mg, twice daily) plus oxcarbazepine (300 mg, twice daily), with good treatment effects. As of the time of this report, the patient had not experienced another seizure for 8 months. Through a series of adjustments to the drug treatment strategy, we have identified the most effective treatment for patients with this type of mutation. This successful practice not only benefits patients with specific diseases but also indicates the feasibility of developing effective treatment strategies for extremely rare or even “unique” mutations, pushing the boundaries of precision medicine to new extremes. Moreover, it provides a valuable methodological framework and instills confidence for the future treatment of other diseases caused by *de novo* mutations or complex rare mutations.

As the present study was retrospective and purely descriptive, it is subject to limitations such as recall and selection biases, and our observations need to be confirmed in a larger cohort. Since the clinical symptoms of NF1 are age-dependent and the patient in our study is still in her teenage years, it remains uncertain whether the patient will develop a more severe phenotype as she ages. We will follow her clinical course closely to improve our understanding of the disease. Experts have long recommended that all children with NF1 should be evaluated annually in a multidisciplinary clinic, with quick and seamless access to the full range of medical subspecialists needed to address the diverse manifestations of this disease (Listernick et al., 2007; Veres et al., 2025). As an autosomal dominant disorder, NF1 is completely episodic, and thus, more attention should be paid to monitoring patients with a family history (Begum-Ali et al., 2025). Prenatal and genetic testing can identify pathogenic *NF1* mutations in both parents but cannot predict the severity of the clinical disease. In addition, not all couples are interested in prenatal testing, meaning that management of the disorder in children and adults requires a collaborative effort between medical professionals and patients. Given the complexity of the disease, the development of effective therapies also requires interdisciplinary collaboration and teamwork.

## 5 Conclusion

The present study reports a *de novo* compound heterozygous mutation that highlights the complexity of the *NF1* gene and the absence of clear genotype–phenotype correlations for many of its known variants, posing challenges for both clinicians and researchers. We analyzed the patient’s family of four using genetic testing, clinical phenotypes, imaging features, and seizure patterns. We also explored potential mechanisms through an in-depth analysis of the patient. Our findings not only broaden the known phenotypic spectrum of *NF1* gene mutations, which can help with the screening and genetic diagnosis of NF1, but also provide new insights that emphasize the importance of other genetic or environmental factors in the development and severity of the disease. Collectively, our findings contribute to a better understanding of the genotypic and phenotypic characteristics of patients with NF1.

## Data Availability

The datasets presented in this study can be found in online repositories. The names of the repository/repositories and accession number(s) can be found in the article/Supplementary material.
