# Supplementary material for: Case Report: Phenotypic heterogeneity within an NF1 family: assessment of the pathogenicity of a de novo c.6640dupA shift mutation and a splice variant with an epilepsy phenotype
Source: Front Neurosci. 2025 Jul 9;19:1604771. doi: 10.3389/fnins.2025.1604771 (PMC12283690; doi:10.3389/fnins.2025.1604771)
Supplement: Supplementary file 1 [file Data_Sheet_1.PDF]

Supplementary Material

1 Supplementary Figures

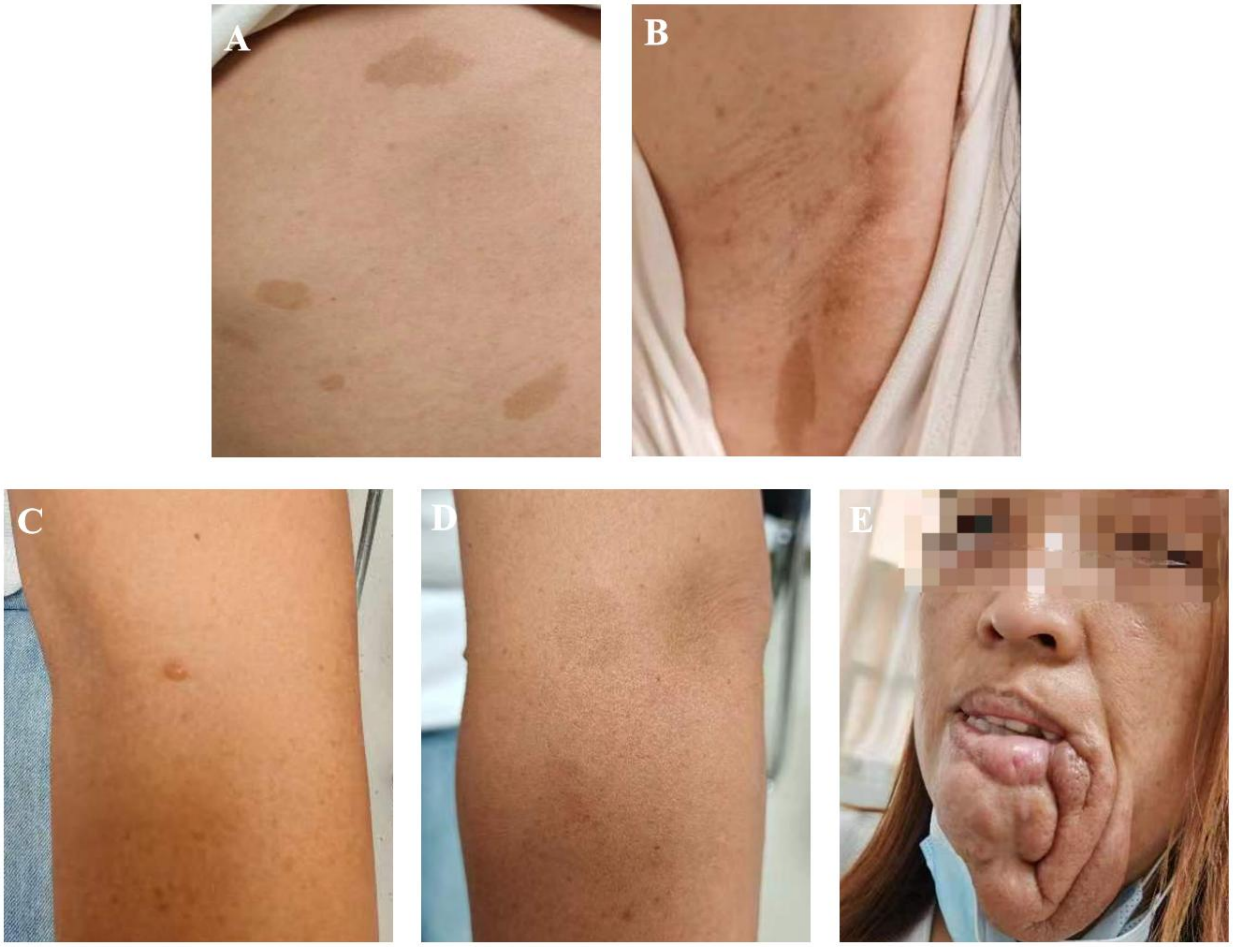

Supplementary Figure 1: A, B The patient had café au lait spots of varying sizes scattered over the trunk. **C, D** The patient's mother had café au lait spots of varying sizes scattered over the trunk. **E** The patient's mother had a hemangioma on her mandible.

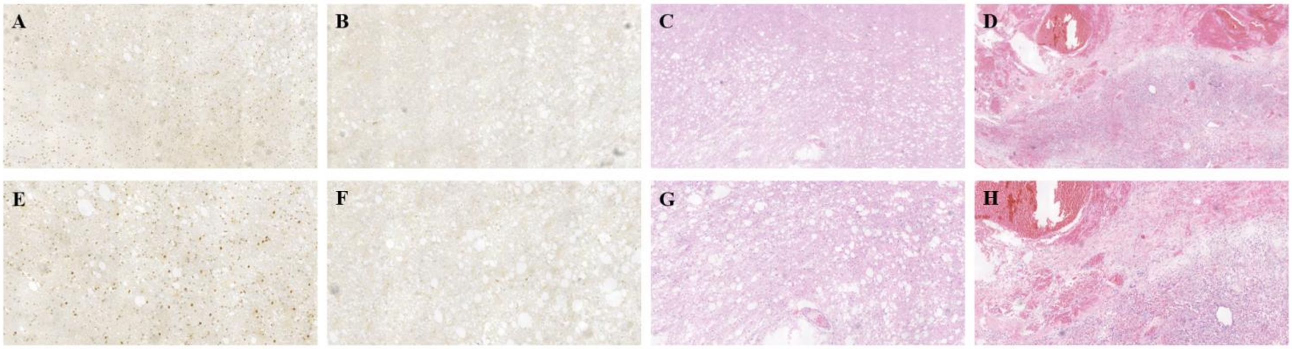

Supplementary Figure 2: Postoperative pathology results from the intracranial tumor of the patient's mother, showing the following: (left temporal lobe) microscopic view of the cerebral cortex and white matter structures, mild disorganization of cortical neuronal structure, glial cell hyperplasia with microcystic formation in part of the cerebral white matter, and hyperplasia of vascular components with lumen of varying sizes in some areas, which was consistent with vascular malformations. Immunohistochemistry: tumor cells were GFAP (+), Olig-2 (+), IDH-1 (-), ATR-X (-), P53 (-), Ki-67 (+, 1%–3%). **A** ATR-X (-) 10×, **B** IDH-1 (-) 10×, **C** microcystic area 4×, **D** vascular area 4×, **E** ATR-X (-) 20×, **F** IDH-1 (-) 20×, **G** microcystic area 10×, and **H** vascular area 10×.

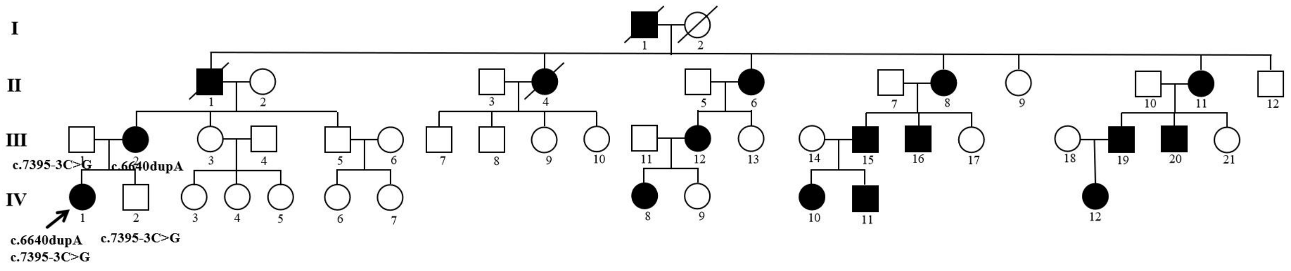

Supplementary Figure 3: Pedigree of the family with NF1. Arrow = proband; square = male; circle = female; strikethrough = deceased; filled symbols = café au lait spots; open symbols = assumed to be healthy.
